# Laryngeal and Subglottic Necrosis Following Long‐Term Heroin Inhalation

**DOI:** 10.1155/crot/5559965

**Published:** 2026-07-13

**Authors:** I. Janssens, G. Vandeplas, M. Maris, B. Ihtijarevic, D. Loose

**Affiliations:** ^1^ Department of Otorhinolaryngology, University Hospital Ghent, Ghent, Belgium, uzgent.be; ^2^ Department of Otorhinolaryngology, Vitaz, Sint-Niklaas, Belgium

## Abstract

While nasal and palatal necrosis secondary to intranasal cocaine use is well documented in the literature, similar presentations linked to heroin abuse are rarely described. To the best of our knowledge, laryngeal involvement has not previously been reported. We present the case of a 48‐year‐old man with a 25‐year history of smoking heroin, who presented with necrotic debris on the vocal cords and in the subglottic region. Debridement was performed, after which the symptoms subjectively improved. After one year, a subglottic adenocarcinoma was diagnosed. Substance abuse, including smoking heroin, should be considered in the differential diagnosis when necrotic laryngeal tissue is observed during laryngoscopic evaluation of patients with dysphonia and dyspnea. Biopsy and debridement are crucial for both diagnosis and symptom management. Long‐term follow‐up is essential, especially in patients with ongoing substance abuse, as malignancy can develop over time.

## 1. Introduction

According to the European Monitoring Center for Drugs and Drug Addiction (EMCDDA), approximately 0.3% of the adult population in the European Union, or around 860,000 individuals, reported using opioids in 2022, with heroin remaining the most commonly used illicit opioid in the region [1]. Heroin is commonly consumed through snorting, smoking, or intravenous injection [1, 2]. A common inhalation technique is “chasing the dragon,” in which heroin is placed on a piece of foil, heating it from below at temperatures exceeding 100°C, and inhaling the vapors through a tube or straw [3]. Notably, routes of heroin administration have shifted significantly in the past decade. The percentage of heroin users who smoke or inhale the drug has significantly increased from 35% in 2013 to 63% in 2022, whereas the rate of injection use has dropped from 37% to 18% during the same timeframe [1, 2]. This transition reflects broader changes in drug use patterns, with smoking or inhaling increasingly favored due to perceptions of reduced health risks compared to injecting [2].

Nasal and palatal necrosis resulting from intranasal cocaine abuse is well‐documented in the literature [4]. However, similar presentations associated with heroin abuse are notably less common and the exact mechanisms by which heroin affects underlying tissues remain poorly understood. While there are limited reports describing nasal or palatal perforations associated with intranasal heroin use or other nonvasoconstrictive substances, laryngeal manifestations of heroin abuse have not, to the best of our knowledge, been previously documented in the literature [5, 6]. Here, we present a unique case of laryngeal necrosis in a patient with a 25‐year history of smoking heroin.

## 2. Case Report

A 48‐year‐old man with a 25‐year history of heroin abuse through inhalation presented to the emergency department where he was referred to the otorhinolaryngology clinic with a history of increased dysphonia and dyspnea since four weeks. No significant dysphagia, odynophagia, or infectious symptoms were reported. The patient denied any usage of cocaine or other recreational drugs. Laboratory findings were noninfectious and negative for autoimmunity. Laryngoscopy showed irregular black debris on both true vocal cords extending to the subglottic region (Figure 1). There was normal bilateral vocal fold motion. No additional upper airway lesions were observed. The patient was of low socioeconomic status and was enrolled in a rehabilitation program, receiving methadone therapy under the supervision of a psychiatrist but was still actively using heroin. The patient also reported smoking a pack of cigarettes a day for 30 years and drinking four units of alcohol each day. No relevant occupational, environmental, or other chemical exposures were reported, and no history of gastroesophageal reflux disease (GERD) was documented. The medical history was significant for lung emphysema, cerebral aneurysms, and Stiff Person Syndrome, a rare autoimmune neurological condition. Family history was negative.

**FIGURE 1:**
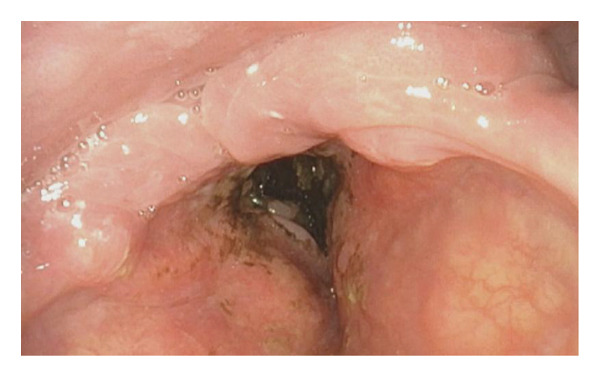
Laryngoscopy showed a black irregular debris on both true vocal cords extending to the subglottic region.

Contrast‐enhanced computed tomography (CT) scan revealed irregular contours of both true vocal cords, accompanied by a mass in the right subglottic region (Figure 2). The lesion measured approximately 1.6 cm anteroposteriorly, 1.1 cm laterally, and 2.3 cm craniocaudally. No evidence of involvement or disruption of the thyroid cartilage was observed. Extensive debridement of necrotic mucosa was performed in the operating room (Figure 3). Biopsy showed necrosis along with numerous neutrophilic polymorphonuclear cells, desquamated squamous cells, and ciliated columnar epithelial cells. Antineutrophil cytoplasmic antibody (ANCA) panel was negative. Diagnosis was noted as heroin‐induced laryngeal necrosis.

**FIGURE 2:**
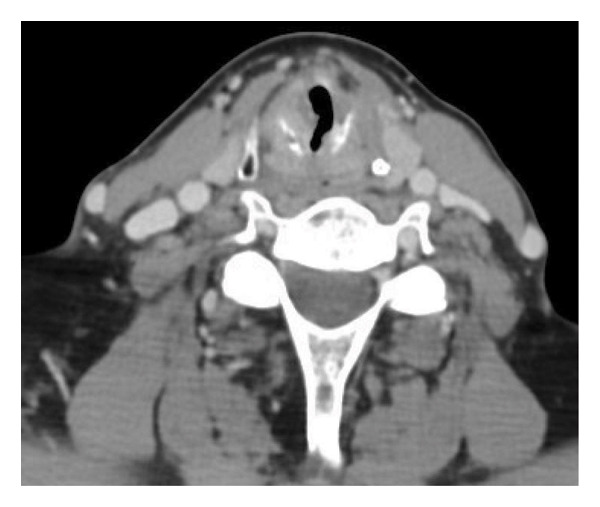
CT scan showed irregular contours of both true vocal cords, accompanied by a mass in the right subglottic region.

**FIGURE 3:**
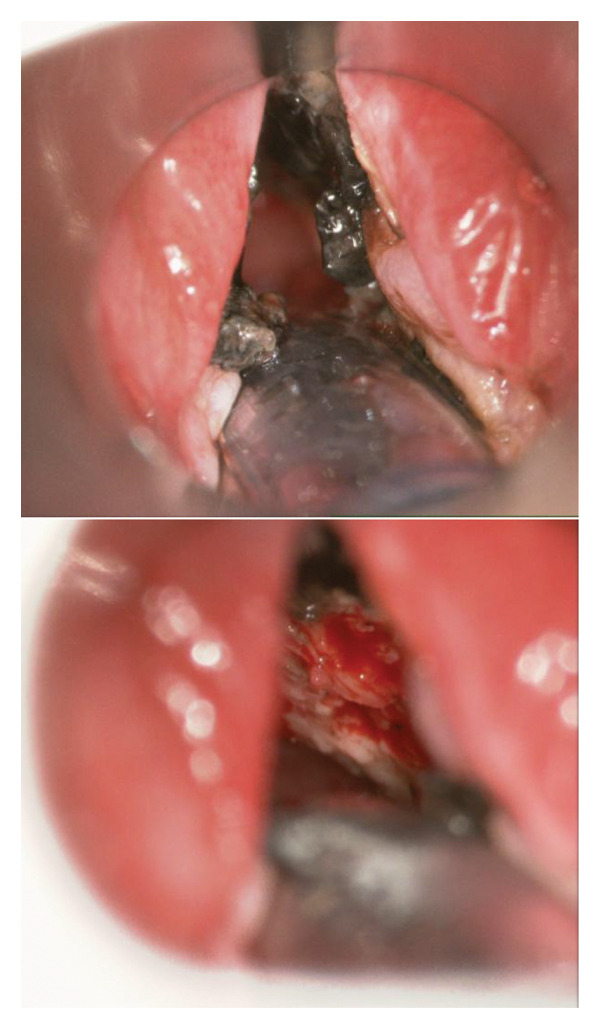
Extensive debridement of necrotic mucosa was performed in the operating room.

Following debridement, the patient reported a significant improvement in breathing. The patient had follow‐up visits every three months over the course of a year. Although he continued to use heroin during this time, both his symptoms and the clinical findings on laryngoscopy remained stable. One year later, due to slight worsening of dysphonia and poor visualization, a new contrast‐enhanced CT‐scan was performed. This CT‐scan showed a contrast‐enhancing lesion in the right subglottic region, extending to the right true vocal cord, with possible invasion into the paralaryngeal soft tissues anteriorly, limited to the strap muscles, and invasion of the cricoid (Figure 4A). Hence, an MRI was performed, which confirmed these findings with a moderate growth of the lesion over the course of one year (Figure 4B). An additional nodule was observed posterobasally against the right thyroid lobe, with a diameter of 1.9 cm, potentially a solitary pathologically enlarged lymph node. An ultrasound‐guided fine needle aspiration cytology (FNAC) of the lesion was performed which showed malignant cells consistent with a poorly differentiated large‐cell carcinoma, with a preference for adenocarcinoma. A biopsy of the suspicious lymph node against the right thyroid lobe was obtained via endobronchial ultrasound guidance and also showed a poorly differentiated large‐cell carcinoma, with a preference for adenocarcinoma. Due to the extent of the disease, the patient is currently being treated with chemoradiotherapy.

**FIGURE 4:**
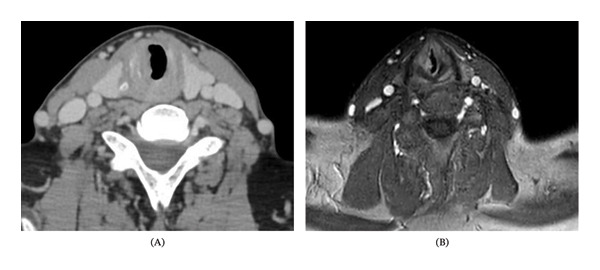
CT (A) and MRI (B) images showed a contrast‐enhancing lesion in the right subglottic region, extending to the right true vocal cord, with possible invasion into the paralaryngeal soft tissues anteriorly, limited to the strap muscles, and invasion of the cricoid.

## 3. Discussion

Nasal and palatal necrosis resulting from intranasal cocaine abuse is well documented in the literature. However, similar presentations due to smoking heroin are less common. To the best of our knowledge, this is the first reported case of laryngeal necrosis extending into the subglottic region possibly due to heroin abuse through inhalation. It has been established that the primary mechanism of action in intranasal cocaine abuse is local vasoconstriction, which leads to ischemia and necrosis when it becomes chronic. However, opioids such as heroin do not induce vasoconstriction, and the pathophysiological mechanisms underlying nasal necrosis and septal perforation remain incompletely understood. One hypothesis states that heroin induces localized immunosuppression, as macrophages and lymphocytes express opioid receptors, leading to the inhibition of key immune mediators such as antibodies and cytokines. This immunosuppression increases susceptibility to infection, with the ensuing inflammatory response potentially contributing to tissue necrosis. An alternative hypothesis proposes that autoimmune degradation is triggered by ANCA [7]. Autoimmune diseases such as granulomatosis with polyangiitis with laryngeal and subglottic involvement were considered in this case, but the ANCA panel was negative. In addition to these proposed mechanisms, thermal injury should be considered as exposure to the high temperatures generated during heroin inhalation by “chasing the dragon” may have contributed to laryngeal mucosal injury and the observed subglottic necrosis [3].

Yufan Lin et al. and Jun Lin et al. both described a similar presentation to this case of fibrinous material lining bilateral true vocal folds and subglottic region as a result of intranasal acetaminophen abuse. Sinus complications from intranasal opioid abuse, with or without acetaminophen, are thought to result from a pronounced inflammatory reaction caused by the drug or its additives [8, 9].

In cases of nasal and palatal lesions, regression of the lesion is observed following the cessation of substance abuse. A similar outcome can be expected in laryngeal presentations. We hypothesize that surgical debridement would have a significant impact on disease progression if the patient were able to abstain from smoking heroin. In the present case, however, the patient was unable to abstain from heroin, tobacco, and alcohol. At the one‐year follow‐up, the progression to malignancy was observed.

While this case highlights severe laryngeal tissue damage, it also prompts consideration of the possible oncogenic properties of opium derivatives such as heroin. The scientific literature presents inconsistent findings regarding a direct causal relationship between opium use and the development of head and neck cancers. However, the International Agency for Research on Cancer (IARC) has classified opium as carcinogenic to humans, with sufficient evidence linking it to cancers of the larynx, lung, urinary bladder, and other sites. The expanding use of opioids, including the inhalation of heroin, together with the emerging recognition of opium’s carcinogenic potential, suggests a critical and complex public health challenge [10–12].

Most laryngeal cancers are squamous cell carcinomas. Primary laryngeal adenocarcinomas are very rare, with an incidence reported as less than 1% of all laryngeal neoplasms. The subglottic adenocarcinoma in this patient should not be attributed to heroin inhalation alone. In addition to chronic tobacco and alcohol use, which are established and potentially synergistic risk factors for laryngeal cancer, GERD and occupational or chemical exposures have also been discussed as possible contributing factors in laryngopharyngeal malignancy [13]. In this case, the relevant documented risk factors were long‐term tobacco use, daily alcohol consumption, and repeated thermal mucosal exposure from high‐temperature heroin inhalation. As this is a single case report, causality between heroin inhalation and the subglottic laryngeal necrosis and adenocarcinoma cannot be established.

## 4. Conclusion

Substance abuse, including heroin inhalation, should be considered in the otorhinolaryngologist’s differential diagnosis when necrotic laryngeal tissue is observed during laryngoscopic evaluation of patients with dysphonia and dyspnea. Biopsy and surgical debridement are essential for accurate diagnosis and symptom relief. Long‐term follow‐up is essential, especially in patients with ongoing substance abuse, as malignancy can develop over time. However, the clinical response should extend beyond oncological vigilance. Providers should use these encounters to support the patient in all aspects of care, including referral to rehab, psychiatric and social support, smoking and alcohol cessation, and structured follow‐up to reduce ongoing exposure.

Although a possible association between opium use and cancer risk has been reported, especially when combined with known risk factors such as tobacco use, there remains a need for large‐scale, longitudinal studies to establish a causal relationship.

## Funding

No funding was received for this manuscript.

## Conflicts of Interest

The authors declare no conflicts of interest.

## Data Availability

The data that support the findings of this study are available from the corresponding author upon reasonable request.

## References

[1] European Monitoring Centre for Drugs and Drug Addiction , Heroin and Other Opioids–The Current Situation in Europe (European Drug Report 2024), 2024, EMCDDA, https://www.euda.europa.eu/publications/european-drug-report/2024/heroin-and-other-opioids_en.

[2] European Monitoring Centre for Drugs and Drug Addiction , Injecting Drug Use in Europe–The Current Situation (European Drug Report 2024), 2024, EMCDDA, https://www.euda.europa.eu/publications/european-drug-report/2024/injecting-drug-use_en.

[3] Strang J. , Griffiths P. , and Gossop M. , Heroin Smoking by ‘Chasing the Dragon’: Origins and History, Addiction. (1997) 92, no. 6, 673–683, 10.1046/j.1360-0443.1997.9266734.x. 9246796

[4] Di Cosola M. , Turco M. , Acero J. , Navarro-Vila C. , and Cortelazzi R. , Cocaine-Related Syndrome and Palatal Reconstruction: Report of a Series of Cases, International Journal of Oral and Maxillofacial Surgery. (2007) 36, no. 8, 721–727, 10.1016/j.ijom.2007.03.015. 17643265

[5] Messinger E. , Narcotic Septal Perforations due to Drug Addiction, JAMA. (1962) 179, no. 12, 964–965, 10.1001/jama.1962.03050120000013. 14472982

[6] Peyrière H. , Léglise Y. , Rousseau A. , Cartier C. , Gibaja V. , and Galland P. , Necrosis of the Intranasal Structures and Soft Palate as a Result of Heroin Snorting: A Case Series, Substance Abuse. (2013) 34, no. 4, 409–414, 10.1080/08897077.2013.781565. 24159913

[7] Zhang D. , Patel K. B. , Cass L. M. , Foster A. E. , Guntupalli L. , and Brunworth J. D. , Heroin-Induced Nasal Necrosis and Septal Perforation, Acta Oto-Laryngologica Case Reports. (2017) 2, no. 1, 145–149, 10.1080/23772484.2017.1379354.

[8] Lin Y. , Lu J. Y. , Pinheiro-Neto C. D. , Jones D. M. , and Gildener-Leapman N. , Intranasal Acetaminophen Abuse and Nasal, Pharyngeal, and Laryngotracheal Damage, Cureus. (2019) 11, no. 8, 10.7759/cureus.5432. PMC6710488 31485385

[9] Lin R. J. and Smith L. J. , Laryngeal Manifestation of Intranasal Acetaminophen Abuse and Review of Literature, Ear, Nose & Throat Journal. (2019) 98, no. 4, 192–194, 10.1177/0145561319836807. 30939912

[10] Bakhshaee M. , Raziee H. R. , Afshari R. , Amali A. , Roopoosh M. , and Lotfizadeh A. , Opium Addiction and Risk of Laryngeal and Esophageal Carcinoma, Iranian Journal of otorhinolaryngology. (2017) 29, no. 90, 19–22. 28229058 PMC5307300

[11] Mohebbi E. , Alimoradi Z. , Rozek L. S. , Mohebbi A. , and Jafari E. , Risk of Head and Neck Cancer Among Opium Users, Archives of Oral Biology. (2024) 157, 10.1016/j.archoralbio.2023.105846. 37980840

[12] Cheraghi Z. , Azmi-Naei B. , Azmi-Naei N. , and Ameri M. , The Significant Impact of Opium Use on Various Types of Cancer: An Updated-Systematic Review and Meta-Analysis, BMC Cancer. (2025) 25, no. 1, 10.1186/s12885-025-13768-y. PMC11863765 40000967

[13] Talamini R. , Bosetti C. , La Vecchia C. et al., Combined Effect of Tobacco and Alcohol on Laryngeal Cancer Risk: A Case-Control Study, Cancer Causes & Control. (2002) 13, no. 10, 957–964, 10.1023/a:1021944123914. 12588092

